# Genetic Diversity of Bovine Pestiviruses Detected in Backyard Cattle Farms Between 2014 and 2019 in Henan Province, China

**DOI:** 10.3389/fvets.2020.00197

**Published:** 2020-04-17

**Authors:** Hongfei Shi, Huan Li, Yang Zhang, Lulu Yang, Yun Hu, Zhicheng Wang, Lisha Duan, Chaoliang Leng, Baolong Yan, Lunguang Yao

**Affiliations:** ^1^Henan Provincial Engineering Laboratory of Insects Bio-reactor, Henan Provincal Engineering and Technology Center of Health Products for Livestock and Poultry, China-UK-NYNU-RRes Joint Libratory of Insect Biology, Nanyang Normal University, Nanyang, China; ^2^Department of Parasitology, School of Basic Medical Sciences, Wenzhou Medical University, Wenzhou, China; ^3^Henan Provincal Engineering and Technology Center of Health Products for Livestock and Poultry, Key Laboratory of Ecological Security and Collaborative Innovation Centre of Water Security for Water Source Region of Mid-line of South-to-North Diversion Project of Henan Province, School of Agricultural Engineering, Nanyang Normal University, Nanyang, China

**Keywords:** *Pestivirus A*, *Pestivirus H*, cattle, genotype, China

## Abstract

Bovine pestiviruses include *Pestivirus A* (BVDV-1), *Pestivirus B* (BVDV-2), and *Pestivirus H*, which was originally called *HoBi-like pestivirus*. We conducted an epidemiological investigation for pestiviruses circulating in backyard cattle farms in central China. RT-PCR assays and sequences analysis were conducted on 54 nasal swabs, 26 serum samples, and three lung samples from cattle with respiratory infections and identified 29 pestivirus strains, including 24 *Pestivirus A* and five *Pestivirus H* strains. Phylogenetic analysis based on partial 5′-UTR and Npro sequences showed that the genotypes of 24 *Pestivirus A* strains included *Pestivirus A* 1b (six isolates), *Pestivirus A* 1m (six isolates), *Pestivirus A* 1q (two isolates), *Pestivirus A* 1u (one isolates), and *Pestivirus A* 1o (nine isolates, a putative new sub-genotype). In addition, a single *Pestivirus H* agenotype included all five *Pestivirus H* strains. This study revealed extensive genetic variations within bovine pestivirus isolates derived from cattle in backyard farms in Central China, and this epidemiological information improves our understanding of the epidemics of bovine Pestiviruses, as well as will be useful in designing and evaluating diagnostic methods and developing more effective vaccines.

## Introduction

Pestiviruses are single-stranded, positive-sense, enveloped RNA viruses with a genome of ~12.3 kb, which belong to the family *Flaviviridae*, genus *Pestivirus*. According to the proposed revision to its taxonomy, the *Pestivirus* genus includes 11 species, namely *Pestivirus A* (*bovine viral diarrhea virus 1*, BVDV-1), *Pestivirus B* (*bovine viral diarrhea virus 2*, BVDV-2), *Pestivirus C* (*classical swine fever virus*, CSFV) and *Pestivirus D* (*border disease virus*, BDV), *Pestivirus E* (*pronghorn pestivirus*), *Pestivirus F* (*Bungowannah virus*), *Pestivirus G* (*giraffe pestivirus*), *Pestivirus H* (*Hobi-like pestivirus*), *Pestivirus I* (*Aydin-like pestivirus*), *Pestivirus J* (*rat pestivirus*), and *Pestivirus K* (*atypical porcine pestivirus*) (1). Among these species, *Pestivirus A, Pestivirus B*, and *Pestivirus H* aroused great concern because these cause significant economic losses in the cattle industry worldwide (2–4). *Pestivirus A* and *Pestivirus B* are major viruses associated with a number of clinical manifestations that range from mild to severe in feedlot cattle, including respiratory disease, digestive disease, and/or reproductive system disturbances and suppression of the immune system (5–7). Natural infections in cattle involving *Pestivirus H* showed similar clinical signs as those of *Pestivirus A* or *Pestivirus B* infections (8–11).

To date, at least 23 genotypes of *Pestivirus A* (12–16) and six genotypes (17) of *Pestivirus B* have been classified based on sequence comparison analyses and the palindromic nucleotide substitutions (PNS) genotyping method (18, 19). *Pestivirus H*, first isolated from fetal calf serum (20), has spread to different continents, including North America, South America, Europe, and Asia (21–26). In China, nine genotypes of *Pestivirus A* (1a,1b, 1c,1d, 1m, 1o, 1p,1q, and 1u) (27–30), two genotypes (2a, 2b) of *Pestivirus B* (31–33), and *Pestivirus H* (24, 34) have been reported. Cattle production by backyard farming is a widespread cattle-keeping pattern in developing countries. In central China, which includes Henan Province, more than 3,720,000 cattle have been raised (35), and previous data showed that over 20% of cattle were kept in small farms (cattle number <10), including a large number of backyard farms in China (36), especially in the southern region of Henan Province, where free-range cattle farms are key economic sectors (35). However, the limited biosecurity measures in these farms usually lead to the introduction and spread of exotic or endemic disease (37–39). Furthermore, in backyard cattle farms in China, most of the animals graze in the wilderness, and thus come into contact with infected cattle. To our knowledge, information on the epidemiology of pestiviruses in cattle in backyard farms in China is limited. The aim of this study was to investigate the distribution of pestiviruses that are associated with respiratory disease from backyard farms in Henan Province, China.

## Materials and Methods

### Samples

From November 2014 to April 2019, a total of 54 nasal swabs and 26 serum samples were collected from different cattle in 41 backyard farms in Henan Province in Central China; these animals had never been vaccinated against *Pestivirus A* and were diagnosed with respiratory infections by rural veterinarians and treated with antibiotics for days, resulting in slow recovery. In addition, three lungs of deceased calves were collected in 2015, 2016, and 2018. All samples were stored at −80°C until analysis.

### Primer Selection

The nested RT-PCR primers for genotyping bovine pestiviruses, including *Pestivirus A, Pestivirus B*, and *Pestivirus H* (40) were used to detect the pestivirus genome in the samples. For phylogenetic analysis, the BVDV-positive samples were further subjected to 5′-UTR and Npro RT-PCR using primers 324/326 (41) and BD1/BD2 (42), respectively. Because the sequences of *Pestivirus H*-positive samples were most closely related to the HN1507 strain (43), the positive samples further subjected to RT-PCR covering a partial 5′-UTR fragment and the entire Npro region with the primers HN-F (sense; 5′-CCTTCAGTAGGACGAGCATAA-3′) and HN-R (antisense; 5′- AGACGGGCTATACCACAATAA-3′), corresponding to nt 109–1,107 of *Pestivirus H* strain HN1507 (GenBank accession number: KU563155).

### RNA Extraction, Amplification, and Sequencing

The three lung samples were first homogenized, then RNA was extracted from the lung homogenates, nasal swabs, and serum samples using an EasyPure Viral DNA/RNA Kit (Transgen Biotech, China) according to the manufacturer's instructions. The RNA was resuspended in DEPC-treated water and kept until analysis. cDNA was synthesized from RNA using Easyscript Reverse Transcriptase kit (Transgen Biotech, China) using random 9-mers as reverse transcription primer.

nRT-PCR to detect the pestivirus genome was performed as described elsewhere (40). Then, the BVDV-positive samples were further subjected to 5′-UTR and Npro RT-PCR earlier described (41, 42). The *Pestivirus H*-positive samples were subjected to RT-PCR in a 50-μL reaction mixture similar to the *Pestivirus A* reaction mixture according using the following conditions: reverse transcription at 50°C for 60 min, then denaturation at 93°C for 3 min; followed by 30 cycles of 94°C for 45 s, 56°C for 45 s, and 72°C for 1 min; and a final extension at 72°C for 10 min. Then the amplified products were recovered from the agarose gel using a gel extraction kit (Omega Bio-Tek, China), and the purified amplicons were directly sequenced in both directions using an ABI automated A373 sequencer (ABI, USA). Lastly, all of the sequences were compared to the NCBI databases using a BLAST search.

### Phylogenetic Analysis

The nucleotide regions of the 5′-UTR were compared and aligned using CLUSTAL W program. Molecular Evolutionary Genetics Analysis version 6 (MEGA6) (44) was used for phylogeny inference according to the neighbor-joining criterion and the Kimura 2-parameter model. The robustness of the hypothesis was tested with 1000 non-parametric bootstrap analyses.

Following strains were used for 5′-UTR Phylogenetic analysis: NADL [M31182] and Singer [L32875] are the references for the Pestivirus A 1a genotype, strains Osloss [M96687], and Draper [L32880] are the references for the Pestivirus A 1b genotype and strains Europa [AB000898], and F [AF298065] are the reference for the Pestivirus A 1.3 genotype. Strains 438/02 [AY159540], PT42-03 [AY944293], 23-15 [AF298059], so CP/75 [AB042661], Shitara-02-06 [LC089876], IS25CP/01 [AB359931], AQGN96B15 [AB300691], Bega [AF049221], Manasi [EU159702], KM [AF298068], G [AF298066], SD0803 [JN400273], isolate 6 [JX276543], 10-84 [AF298054], 3186V6 [AF298062], 11207/98 [AJ304390], 22146/81 [AJ304376], 2561 [JQ920287], 17P [AF244954], KS86-1ncp [AB042713], Deer [AB040132], TR70 [MG670547], TR75 [MG670549], ZM-95[AF526381], TJ0801 [GU120255], BJ1305 [KF925505], XZ-24 [KJ578918], TR-2007-Gu-175454-4695 [EU716150], TR16 [MG670548], TR72 [MG670546], J [AF298067], W [AF298073], BJ0702 [GU120248], BJ0703 [GU120249], A [AF298064], L [AF298069], CH-01-08 [EU180024], 71-03 [KF205294], PG/13a/07 [not deposited], GXBH-EB34 [KJ578813], GXLZ-BB4 [KJ578814], 130/15-4215 [KY085998], 130/15-5364 [KY085999], Rebe [AF299317], SuwaCp [AF117699], SuwaNcp [AF117700], CH-05-b1 [EU180030], and S153 [KF006964] are references for the Pestivirus A 1.4 to Pestivirus A 1.23.

Following strains were used for Npro Phylogenetic analysis: NADL [M31182], Oregon C24V [AF091605] and SD-1 [M96751] are the references for the Pestivirus A 1a genotype, strain Osloss [M96687] is the reference for the Pestivirus A 1b genotype. Strains F [AF287284], 10JJSKR [KC757383], 23/15 [AF287279], 58-1 [KF023454], 2541 [JQ920342], so CP/75 [AB105590] are references for the Pestivirus A 1.3, 1.5 and 1.6 genotypes. Strains IS25CP/01 [AB359931], IS26NCP/01 [AB359932], Bega [AF049221], 519 [AF144464], Deer-NZ1 [U80903], G [AF287285], CH-SM09/20 [AY895007], SD0803 [JN400273], isolate 6 [KC207072], 3186V6 [AF287282] and 26-V639 [AF287282] are references for the Pestivirus A 1.7 to Pestivirus A 1.11 genotypes. Strains Deer-GB1 [U80902] and KS86-1ncp [AB078950] are references for the genotypes Pestivirus A 1.13. Strains TR70 [KF154779], TR73 [KF154777] and TR75 [KF154778], reported as genotype R (Yesilbag et al., 2014), are references for genotype 1.14. Strains BJ1305 [KF925522], TJ0801 [GU120262] and ZM-95 [AF526381] are references for the genotype Pestivirus A 1.15. Strains TR16 [EU163964], TR27 [EU163975], TR29 [EU163977] and TR72 [KF154776] are references for the genotype Pestivirus A 1.16. Strains J [AF287286], W [AF287290], BJ0701 [GU120259], BJ0702 [GU120260], BJ0703 [GU120261], A [AF287283], L [AF287287], CH-01-08 [EU180033], 71-03 [KF205326], M31182 [JQ799141], 441/09 [KY040435], CH-Bohni [AY894997] and CH-Suwa [AY894998] are references for the Pestivirus A 1.17 to Pestivirus A 1.22 genotypes.

## Results

**Figure 1:**
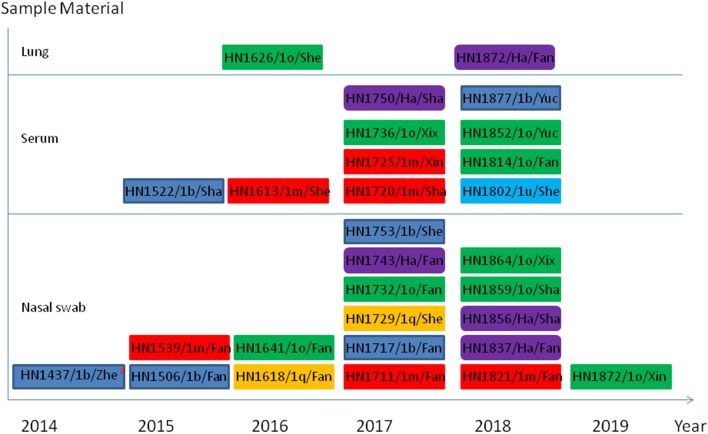
Schematic representation of 29 bovine *Pestivirus* positive in this work. ^a^indicate the information of sample including sample number/genotype/county. The county names: Zhe, Zhenping; Fan, Fangcheng; Sha, Shangcai; She, Sheqi; Xin, Xinye; Xix, Xixia; Yuc, Yucheng. The blue rectangle represents *Peativirus A* 1b genotype; the red rectangle represents *Peativirus A* 1m genotype; the yellow rectangle represents *Peativirus A* 1q genotype; the green rectangle represents *Peativirus A* 1o genotype; the light blue rectangle represents *Peativirus A* 1u genotype; the purple rounded rectangle represents *Peativirus H* a genotype.

Using first-round nRT-PCR to identify bovine pestivirus by amplification of a 1,013-bp fragment, 83 samples were screened and the pestivirus genome was detected in 17 out of the 54 nasal swab samples, 10 out of the 26 serum samples, and two out of three lung samples. The results of the second-round nRT-PCR showed that in 17 positive nasal swab samples, 14 were positive for *Pestivirus A* and the other three were positive for *Pestivirus H*; in the 10 positive serum samples, nine were positive for *Pestivirus A* and one was positive for *Pestivirus H*; in the two positive lung samples, one was positive for *Pestivirus A* and the other was positive for *Pestivirus H*; no *Pestivirus B*-positive samples were detected. The 29 pestivirus-positive samples are presented in Figure 1.

The sequences detected by 5′-UTR and Npro RT-PCR in 24 BVDV-positive samples were deposited in GenBank under accession numbers: MN442360–MN442383 and MN442389–MN442412. Sequence alignment of the 5′-UTR and Npro region of the 24 samples using CLUSTAL W indicated a sequence identity within the range 82.0–100% and 70.0–99.8%, respectively. BLAST analysis of the 5′-UTR and Npro sequences showed that all 24 BVDV-positive isolates belonged to *Pestivirus A*. The comparative analysis among Henan isolates and the reference strains of *Pestivirus A* (NADL, VEDEVAC) shared a 5′-UTR and Npro region sequence identity within the range 82.9–98.8%, 72.2–97.6%, respectively.

The sequences detected by 5′-UTR and Npro RT-PCR in the five *Pestivirus H*-positive samples were deposited in GenBank as accession numbers MN442384–MN442388 and MN442413–MN442417. Sequence alignment using CLUSTAL W program of the five samples revealed a 5′-UTR and Npro region respective sequence identity within the range 95.8–99.5% and 98.4–99.6%. BLAST analysis of the 5′-UTR and Npro sequences showed that all five *Pestivirus H*-positive isolates belonged to *Pestivirus H*. Comparative analysis of the 5′-UTR and Npro region of the Henan isolates and the reference strains of *Pestivirus H* (TH/04_KhonKaen, HN1507) revealed a sequence identity within the range of 87.5–99.5% and 90.3.2–99.4%, respectively. In particular, the nucleotide homologies between these isolates and the other strain (HN1507) (24, 43) isolated from goat in the same area were 97.9±1.6% and 98.6 ± 0.4% in the above two regions.

**Figure 2:**
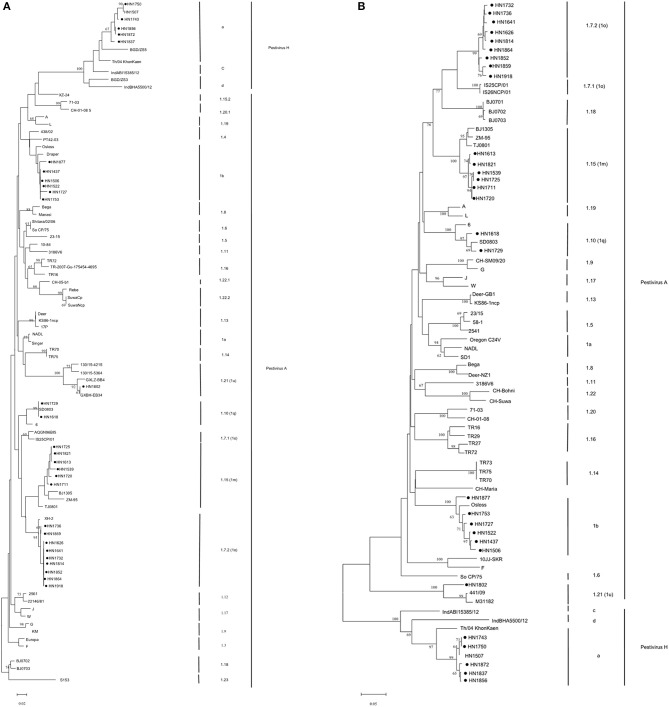
Phylogenetic analysis of bovine *Pestivirus* from cattle in backyard farms in Central China, and reference strains using the 5′-UTR **(A)** and Npro **(B)** sequences. Bootstrap analyses that were supported by >60% of 1,000 replicates are indicated in nodes. Henan pestivirus isolates in this work are highlighted with a symbol (•).

All 24 isolates from the BVDV-positive samples were classified as *Pestivirus A*, and on the basis of phylogenetic analysis of 5′-UTR and Npro genes (Figure 2) further classified into five genotypes: *Pestivirus A* 1b (six isolates), *Pestivirus A* 1m (six isolates), *Pestivirus A* 1q (two isolates), *Pestivirus A* 1u (one isolate), and the other nine *Pestivirus A* isolates cluster in the same genotype with Chinese strain XH-2 which was assigned as *Pestivirus A* 1o, but phylogenetic analysis showed this cluster of isolates were under different cluster from other *Pestivirus A* 1o strains, further analyzed by the PNS software which available at www.pns-software.com (45), these cluster should be members of a new sub-genotype (1.7.2) within the genotype 1o (1.7). In addition, five isolates from *Pestivirus H*-positive samples were classified as *Pestivirus H*, and all further were classified into genotype “*Pestivirus H* a” based on the results of phylogenetic analysis of the 5′-UTR and Npro genes (46) (Figure 2).

## Discussion

China is one of the countries that have the largest domesticated ruminant population in the world, including a large number of backyard farms (35). In 2017, commercial *Pestivirus A or Pestivirus B* vaccines were released to the market. However, vaccination is not mandatory, and awareness of the importance of immunization to prevent Pestivirus infections among backyard farm keepers is rare, despite the occurrence of pestivirus epidemics in large-scale farms in China in recent years (27–29, 47). It is thus essential to investigate the genetic diversity of pestiviruses in backyard farms. Furthermore, the new genotype pestivirus might result in the immune failure of pestivirus vaccine (48). For these above reasons, the genetic diversity of 29 pestivirus-positive samples derived from infected calves in backyard farms from central China was investigated by phylogenetic analysis of the 5′-UTR and Npro partial genomic regions.

The results of phylogenetic analysis showed that *Pestivirus A*−1o, *Pestivirus A*−1b, and *Pestivirus A*−1m were the predominant genotypes in our samples, followed by *Pestivirus A*−1q and *Pestivirus A*−1u. Furthermore, among these three predominant genotypes, *Pestivirus A*−1b and *Pestivirus A*−1m are frequently reported in China (27–30, 49, 50) the *Pestivirus A* 1a found in Henan province (51) was not detected in this work.

Six isolates of the *Pestivirus A* 1b genotype were detected in Zhenping, Fangcheng, Shangcai, Sheqi, and Yucheng in this study (Figures 1, 2). This widespread distribution of *Pestivirus A* is not surprising, as the first genotype of *Pestivirus A* was isolated in China in 1983. Subsequently, *Pestivirus A*−1b was later detected in most of the provinces, including Henan Province and the neighboring provinces numerous reports (27–29, 47, 49, 52–55). Meanwhile, a recent study has shown that 31.6% (2193:6939) of the corresponding *Pestivirus A* isolates around the world were *Pestivirus A*−1b (16). These reports indicate the strong spreading ability of *Pestivirus A*−1b among large-scale farms, and the high detection rate (6/24) in our study also suggests that *Pestivirus A*−1b is a predominant genotype among backyard farms.

Six isolates of the *Pestivirus A* 1m genotype were detected in Fangcheng, Sheqi, Shangcai, and Xinye in this study (Figures 1, 2). The first *Pestivirus A*−1m strain ZM-95 in China was isolated from pigs in 1995 (56). Subsequent reports revealed that *Pestivirus A*−1m is emerging in most of provinces and is considered to be a predominant *Pestivirus A* genotype in herds (27, 30, 50, 54, 57), and also detected in Henan province and neighbor province (29). In addition, sequences of *Pestivirus A*−1m strains in different regions showed high-nucleotide homology, indicating that these strains share the same origin (27, 49). Recently, other surveys on goats uncovered that the *Pestivirus A*−1m could infect goats naturally and cause diarrhea (58). In this study, *Pestivirus A* 1m strains were detected in different backyard farms that shared grassland with goats, this feeding method provided more chances for interspecies transmission of BVDV-1m, further accelerating the evolution of these viruses and more widely spreading disease.

The other nine *Pestivirus A* isolates shared the highest sequence identities (97–98%) in strains such as XH-6, XH-5, XH-1, XH-2, and BJ09 that were isolated from other provinces in China which was assigned as *Pestivirus A* 1o. Furthermore, the Npro sequences of these strains were not found in GenBank, and thus, confirmation could not be done based on the Npro phylogenetic tree analysis. The Npro sequences of the 21 isolates were also analyzed by BLAST, and the highest identities (86–91%) were observed in strain IS26/01ncp from Japan, and BJ0703, BJ0702, BJ0701, and JS12/02 that were isolated from other provinces including the Jiangsu province near to the Henan province in China and were classified as BVDV-1o or BVDV-1p (27, 58, 59). Then the PNS method was used to analyze the existing strain such as XH-2, then the genotype *Pestivirus A* 1.7 (1o) was verified, but the cluster in phylogenetic tree this cluster was in different cluster from other *Pestivirus A* 1.7 (1o) strains, this result indicated these strains form a new sub-genotype (1.7.2). (Figures 2A,B). *Pestivirus A* 1o was first isolated from a calf that developed a mucosal disease and from PI calves in Japan (60), and has been detected in camels, goats, and pigs in China (30, 50, 58). In this study the new sub-genotype *Pestivirus A* 1o were detected in different backyard farms in a few of counties, and the *Pestivirus A* 1o could infect goats and sheep, it is in need that necessary measures should be taken to avoid this new sub-genotype *Pestivirus A* 1o spread in other hosts.

Five isolates of the *Pestivirus H* “a” genotype were detected in Fangcheng and Shangcai in this study (Figures 1, 2). Before this research, Pestivirus H has been previously detected in goats and sheep in Fangcheng (24), and phylogenetic analysis showed that these five isolates were closely related to the HN1507 strain isolated from goat (43). These results indicated that these isolates shared the same origin. Furthermore, considering that these strains were all isolated from animals raised in backyard farms where livestock commonly grazed in the mix, this specific feeding method provides a convenient route for interspecies transmission of *Pestivirus H*. To date, in China, *Pestivirus H* has been reported in contaminated cells, commercial FCS, goats, and sheep (24, 34, 61). This study showed that *Pestivirus H* could be detected in cattle immediately after being detected in goats and sheep in Central China (24).

Bovine pestivirus isolates in backyard farms exhibited a high level of genetic diversity, as indicated in the novel epidemic genotypes of *Pestivirus A* and *Pestivirus H* first emerging in cattle in China. These results indicate that backyard cattle farms could be a special reservoir for the evolution of bovine Pestivirus and provide an important complement to understand the epidemics of bovine Pestivirus. Furthermore, this study will be useful in designing and evaluating diagnostic methods and in developing more effective vaccines.

## Conclusion

Several genotypes of *Pestivirus A* and *Pestivirus H* infections were identified in cattle with respiratory diseases and kept in backyard farms by RT-PCR, sequencing, and phylogenetic analysis. This is the first report on the molecular evidences on natural infections of *Pestivirus H* in cattle in China.

## Data Availability Statement

Datasets are in a publicly accessible repository: The datasets generated for this study can be found in GenBank: https://www.ncbi.nlm.nih.gov/genbank/. The Genbank accession numbers are mentioned in the Results of the article.

## Ethics Statement

The processes of nasal swabs and blood from cattle were approved by their hosts, and all lungs were from animals found dead in the study. The study was approved by the Animal Welfare and Ethics Committee of Nanyang Normal University (No 14027).

## Author Contributions

HS participated in the design of the study, and drafted the main parts of the manuscript. HL, YZ, and LYan participated in the sample collection and PCR detection. YH, ZW, LD, and CL participated in the data analyzing. BY and LYao participated in revised the manuscript and supervised the project.

## Conflict of Interest

The authors declare that the research was conducted in the absence of any commercial or financial relationships that could be construed as a potential conflict of interest.
